# Targeting Beclin1 as an Adjunctive Therapy against HIV Using Mannosylated Polyethylenimine Nanoparticles

**DOI:** 10.3390/pharmaceutics13020223

**Published:** 2021-02-06

**Authors:** Myosotys Rodriguez, Yemmy Soler, Mohan Kumar Muthu Karuppan, Yuling Zhao, Elena V. Batrakova, Nazira El-Hage

**Affiliations:** 1 Department of Immunology and Nanomedicine, Florida International University, Herbert Wertheim College of Medicine, Miami, FL 33199, USA; myrodrig@fiu.edu (M.R.); ysole002@fiu.edu (Y.S.); mmuthuka@fiu.edu (M.K.M.K.); 2 UNC Eshelman School of Pharmacy, University of North Carolina at Chapel Hill, Chapel Hill, NC 27599, USA; yulingz@email.unc.edu (Y.Z.); batrakova@unc.edu (E.V.B.)

**Keywords:** Beclin1, intranasal delivery, in vivo imaging system, polyethylenimine nanoparticle, HIV

## Abstract

Using nanoparticle-based RNA interference (RNAi), we have previously shown that silencing the host autophagic protein, Beclin1, in HIV-infected human microglia and astrocytes restricts HIV replication and its viral-associated inflammatory responses. Here, we confirmed the efficacy of Beclin1 small interfering RNA (siBeclin1) as an adjunctive antiviral and anti-inflammatory therapy in myeloid human microglia and primary human astrocytes infected with HIV, both with and without exposure to combined antiretroviral (cART) drugs. To specifically target human microglia and human astrocytes, we used a nanoparticle (NP) comprised of linear cationic polyethylenimine (PEI) conjugated with mannose (Man) and encapsulated with siBeclin1. The target specificity of the PEI-Man NP was confirmed in vitro using human neuronal and glial cells transfected with the NP encapsulated with fluorescein isothiocyanate (FITC). PEI-Man-siBeclin1 NPs were intranasally delivered to healthy C57BL/6 mice in order to report the biodistribution of siBeclin1 in different areas of the brain, measured using stem-loop RT-PCR. Postmortem brains recovered at 1–48 h post-treatment with the PEI-Man-siRNA NP showed no significant changes in the secretion of the chemokines regulated on activation, normal T cell expressed and secreted (RANTES) and monocyte chemotactic protein-1 (MCP-1) and showed significant decreases in the secretion of the cytokines interleukin 6 (IL-6) and tumor necrosis factor alpha (TNF-α) when compared to phosphate-buffered saline (PBS)-treated brains. Nissl staining showed minimal differences between the neuronal structures when compared to PBS-treated brains, which correlated with no adverse behavioral affects. To confirm the brain and peripheral organ distribution of PEI-siBeclin1 in living mice, we used the In vivo Imaging System (IVIS) and demonstrated a significant brain accumulation of siBeclin1 through intranasal administration.

## 1. Introduction

With the blood–brain barrier (BBB) being a major obstacle for the entry of a drug into the brain, intranasal drug delivery has emerged as a reliable method to bypass the BBB and treat neurological diseases. The composition of the BBB, together with the presence of efflux pumps and enzymes, obstruct the entry of many drugs, including combination antiretroviral therapy (cART), into the brains of HIV-infected individuals [1,2]. In addition, cART is not completely effective in controlling HIV replication in brain reservoirs (including microglia and, to a lesser degree, astrocytes) and does not directly target the inflammatory cascades that are believed to be the primary cause of neuronal injury or dysfunction related to HIV-associated neurological pathology [3,4,5]. Considering these limitations, many alternative methods to developing anti-HIV drugs are under investigation [1,6,7,8,9]. Intranasal delivery is an emerging approach to deliver drugs directly to the brain, bypassing the BBB, and unlike the parenteral route, intranasally administered drugs avoid elimination by the liver, kidney filtration, gastrointestinal tract, and serum degradation. To improve drug delivery to the brain while minimizing tissue damage, methods to bypass the BBB via alternative administration routes—particularly noninvasive intranasal delivery [10,11,12]—have shown positive results with biomolecules and as a potential option to deliver therapeutic agents to target cells in the brain. Direct contact with the nasal epithelium allows the transport of molecules via olfactory- and trigeminal-associated extracellular pathways to directly enter into the central nervous system (CNS) [13,14,15]. Intranasal transmission to the brain has shown great success in drug delivery in several human clinical trials [16,17,18].

The use of nanotechnology for diagnostic and therapy has recently gained substantial popularity. Metallic, organic, inorganic, and polymeric nanostructures have been widely used to target different organs for drug delivery [19,20]. The linear cationic polymer polyethylenimine (PEI) possesses nucleotide binding and condensing activity, together with a high pH buffering capacity that is believed to protect DNA/RNA from degradation and to enhance exit from the endosomal compartment [21,22,23]. Accordingly, PEI is effective in gene delivery into a variety of cell types, even without the addition of cell-binding ligands or endosomolytic agents. We have previously shown the in vitro efficacy of a cationic linear PEI-Beclin1 siRNA nanoparticle (NP) in attenuating HIV replication and reduced the secretion of viral-induced inflammatory molecules in glial cells (microglia and astrocytes) [24]. Beclin1 is an essential protein in regulating the autophagy pathway, a lysosomal degradation pathway that engulfs and sequesters cytoplasmic proteins and other damaged organelles using a unique membranous compartment of the autophagosome. The modulation of autophagy is proposed as a therapeutic avenue for various diseases, including cancer, diabetes mellitus, cardiovascular diseases, and neurodegeneration [25,26,27,28,29].

Here, we confirmed both an anti-inflammatory and an antiviral response of Beclin1 small interfering RNA (siBeclin1) as an autophagy-based therapy in HIV-infected human microglia and human astrocytes and as an adjunctive therapy in combination with cART. We also synthesized a siBeclin1-PEI NP conjugated with mannose (PEI-Man) particles to specifically target mannose-expressing brain cells in vitro and in vivo via intranasal delivery in C57BL/6 mice.

## 2. Materials and Methods

### 2.1. Cell Culture and HIV Infection

Commercially procured human microglia cell line (CRL-3304) from the American Type Culture Collection (ATCC^®^, Manassas, VA, USA), primary human astrocytes (Cat#: 1800) and primary human neurons (Cat#: 1520) from ScienCell Research Laboratories (Carlsbad, CA, USA) were grown to ~75–80% confluency and cultured as per the manufacturer’s protocols. Human microglia were infected with the HIV_SF162_ strain (1 ng/mL) for 7 days. Cells were exposed to either 0.1% DMSO as the vehicle control or 10 μM of the antiviral nucleoside reverse transcriptase inhibitor (NRTI) emtricitabine (FTC) or abacavir (ABC), the protease inhibitors lopinavir (LPV) or atazanavir (ATV), or the integrase inhibitor raltegravir (RGV), with or without siBeclin1 (4 μg). In separate experiments, human microglia and human astrocytes were infected with the HIV_SF162_ strain (1 ng/mL) for 7 days, followed by exposure with a combination of emtricitabine (10 μM), ritonavir (10 μM), and atazanavir (10 μM), hereafter named as cART-1, or lopinavir (10 μM), abacavir (10 μM), and raltegravir (10 μM), hereafter named as cART-2. Combined ART were added with or without Beclin1 siRNA (siBeclin1) (4 μg) (Cat#: sc-29797, Santa Cruz Biotechnology, Santa Cruz, CA, USA). Antiretroviral drugs were provided by the NIH AIDS Reagent Program and reconstituted in DMSO.

### 2.2. Transfection of siRNA into Microglia, Astrocytes, and Neurons

Human microglia, astrocytes, and neurons were transfected with Beclin1 siRNA (Cat#: sc-29797) or fluorescein isothiocyanate (FITC) siRNA (Cat#: sc-36869) purchased from Santa Cruz Biotechnology. The transfection reagent, a mannosylated PEI reagent, was purchased from Polyplus-transfection (New York, NY, USA) and used according to the manufacturer’s protocols.

### 2.3. Immunochemistry

Human microglia, astrocytes, and neurons were fixed in 4% paraformaldehyde, permeabilized with 0.1% Triton X-100, blocked in 10% milk/0.1% goat serum, and immunolabeled with anti-Glial fibrillary acidic protein (GFAP) antibody (Cat#: ab5804, Millipore, Bedford, MA, USA) at a 1:1000 dilution, anti-Ionized calcium-binding adaptor molecule 1 (Iba1) (Cat#: sc32725, Santa Cruz Biotechnology) at a 1:100 dilution, anti-Microtubule-associated protein 2 (MAP2) (Cat#: MAB378, Millipore, Bedford, MA, USA) at a 1:200 dilution, and anti-Mannose receptor (Cat#: ab195192, Abcam, Cambridge, MA, USA) at a 1:100 dilution. Immunoreactivity was visualized with secondary antibodies from Molecular Probes (Carlsbad, CA, USA). 4′,6-Diamidino-2-phenylindole (DAPI) staining was used to label cell nuclei. Images were acquired using a Zeiss (Oberkochen, Germany) inverted fluorescence microscope with a 560 Axiovision camera.

### 2.4. Time-Lapse Assessment of Neuronal Viability

Time-lapse digital images of human neurons were recorded using an inverted microscope with an automated computer-controlled stage encoder and environmental chamber (37 °C, 95% humidity, 5% CO_2_) (Zeiss) that allowed repeated tracking of individual neurons per treatment over time. Neuronal death was considered to have occurred upon collapse and fragmentation of the cell body.

### 2.5. Viability Assay

Viability of each brain cell type was assessed using a live/dead cell fluorescence assay, which combined fluorescent reagents to yield two-color discriminations of the population of live cells, indicated by green fluorescence, from the dead cell population, indicated by red fluorescence (ScienCell Research Laboratories). Brain cells were imaged using an inverted fluorescence microscope (Zeiss), and viable cells were manually quantified and reported as a percent of viability.

### 2.6. Animals

C57BL/6 mice (stock # 000664) were procured from The Jackson Laboratory (Bar Harbor, ME, USA) and bred in the animal facility at Florida International University, Miami, Florida and in the animal facility at the University of North Carolina, Chapel Hill, North Carolina. All animal experiments were carried out in accordance with the approved IACUC protocol 18-007-CR02 issued by Florida International University and the IACUC protocol #19-092.0-A issued by the University of North Carolina.

### 2.7. Intranasal Administration of siRNA-PEI Nanocomplex into C57BL/6 Mice

Beclin1 siRNA (siBeclin1) (Cat#: sc-29797, Santa Cruz Biotechnology) targets the *BECN1* gene involved in the autophagy pathway in murine cells. siRNA are gene silencers that consist of pools of three-to-five target-specific 19–25 nucleotide sequences. Beclin1 siRNA or phosphate-buffered saline (PBS) were administered intranasally in healthy adult C57BL/6 mice using the mannosylated PEI (PEI-Man) (Cat#: 203-10G, Polyplus-transfection, New York, NY, USA) according to the manufacturer’s protocols and as previously described [24]. Animals (*N* = 12/group) received 20 μL of PBS, 20 μL of 8 μg/kg (*N/P* = 8), 20 μL of 10 μg/kg (*N/P* = 8), or 20 μL of 20 μg/kg (*N/P* = 8) of PEI-Man-siBeclin1 via the intranasal route into each nostril. After the indicated time points, mice were sacrificed, and brains recovered were frozen in liquid nitrogen. Half of the brain hemisphere was used for histology and immunofluorescence analysis, and the other hemisphere was minced and used for biochemical analysis.

### 2.8. Reverse Transcription Polymerase Chain Reaction (RT-PCR)

siBeclin1 concentrations in the brain and lung tissues were measured by stem-loop RT-PCR using a TaqMan™ MicroRNA Reverse Transcription Kit (Cat#: 4366596, Life Technologies, Carlsbad, CA, USA) and customized primers for both the TaqMan^®^ qPCR assay and the specific stem-loop RT primer for the target siRNA sequence (Cat#: 4398987, Life Technologies), according to the manufacturer’s protocols.

### 2.9. Nissl Staining

Postmortem brain tissues were cryopreserved in 4% paraformaldehyde and by serial exposure to 10% and 20% sucrose, followed by embedding in Tissue Tek optimal cutting temperature compound (Sakura Finetek, Torrance, CA, USA). Brain sections of 10-micron thickness were stained with Cresyl violet acetate solution (Nissl). Briefly, sections were rewarmed at room temperature for 30 min, then exposed to xylene and immersed in 100% ethanol, 95%, 75% ethanol, and distilled water and then stained with a Cresyl violet solution for 20 min. Sections were washed in distilled water, immersed in 75% ethanol, 95%, and 100% ethanol. Tissues were then cleared by xylene and mounted using mounting media for visualization. Representative images are shown at 20× and 40× magnification.

### 2.10. Enzyme-Linked Immunosorbent Assay (ELISA)

Brains recovered postmortem were minced in RIPA lysis buffer and used to measure the levels of tumor necrosis factor alpha (TNF-α), interleukin (IL)-6, monocyte chemotactic protein-1 (MCP-1), and regulated on activation, normal T cell expressed and secreted (RANTES) by ELISA (R&D Systems, Minneapolis, MN, USA), according to the manufacturer’s instructions. The optical density (O.D.) was read at A450 on a Synergy HTX plate reader (BioTek, Winooski, VT, USA).

### 2.11. Liposome Composition and In Vivo Imaging System (IVIS)

PEI-siBeclin1 complexes at a *N/P* ratio of 8 were generated using mannosylated PEI (PEI-Man) (Polyplus-transfection) according to the manufacturer’s protocols and as previously described [24]. The anionic liposome composed of L-α-phosphatidylserine/L-α-phosphatidylcholine/cholesterol/1,2-distearoyl-sn-glycero-3-phosphoethanolamine-N-(dibenzocyclooctyl), at molar ratios of 20/49/30/1, respectively, was commercially procured from CD Bioparticles (Shirley, NY, USA). To visualize the liposomes, their lipid membranes were labeled with a hydrophobic dye, DIR (DiIC18(7); 1,1′-dioctadecyl-3,3,3′,3′-tetramethylindotricarbocyanine iodide). Briefly, the anionic liposome with a size of 100 nm in diameter was labeled with the hydrophobic fluorescent dye, DIR, in phosphate-buffered saline at a pH of 7.4 and further encapsulated with and without the PEI-Man-siBeclin1. The zeta potential was determined by dynamic light scattering using a Nano ZS90 Zetasizer (Malvern Instruments, Malvern, UK), as previously described [30]. NPs were washed using distilled water by centrifugation and used for in vitro and in vivo release. Lipid NPs (3 × 10^12^ particles/mL) were injected into mice through intranasal administration (20 μL). The biodistribution of NPs intranasally administered in C57BL/6 mice (*N* = 4) were imaged by Xenogen IVIS Optical Imaging System (Spectral Instrument Imaging, Tucson, AZ, USA). To establish background fluorescence levels, animals were imaged before the NP administration. At the endpoint (96 h), animals were sacrificed, and perfused brain and other organs were removed, washed, post-fixed in 10% phosphate-buffered paraformaldehyde, and evaluated by IVIS Aura software (Spectral Instrument Imaging, Tucson, AZ, USA). The background fluorescent level (approximately 0.2–0.3 × 10^5^ RFU) were subtracted from the levels at each time point for each mouse and plotted versus time.

### 2.12. Statistical Analysis

Data were analyzed using analysis of variance (ANOVA) techniques followed by the appropriate post hoc test for multiple comparisons when necessary (GraphPad Prism 7 software, La Jolla, CA, USA). A value of *p* < 0.05 was considered significant.

## 3. Results

### 3.1. siBeclin1 Reduces Viral Production in HIV-Infected Human Microglia Co-Administered with Antiretroviral Drugs and Attenuates Secreted Viral-Induced Inflammatory Molecules in Human Microglia and Human Astrocytes

**Figure 1:**
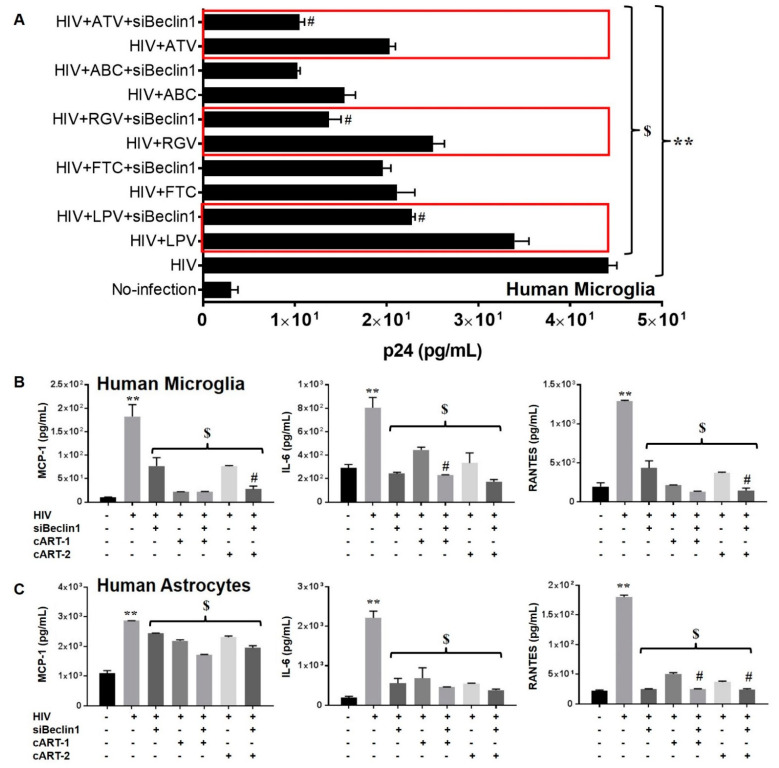
Quantitative measures of viral replication and inflammatory molecules in glia. HIV replication in human microglia was measured using HIV p24 Gag protein ELISA (**A**). Values were determined from standard curves and are presented as the mean ± the standard error of mean (SEM) of three independent experiments. Corresponding cell culture supernatants from human microglia (**B**) and human astrocytes (**C**) were used to detect the levels of monocyte chemotactic protein-1 (MCP-1), interleukin 6 (IL-6), and regulated on activation, normal T cell expressed and secreted (RANTES) by ELISA (**B**,**C**). Values were determined from standard curves and are presented as the mean ± the SEM of three independent experiments (*p* < 0.05; ** vs. media, $ vs. HIV, and # vs. respective antiretroviral).

We determined the efficacy of siBeclin1 as an adjunctive therapy in combination with antiretrovirals in vitro. The antiretrovirals were chosen based on their mechanism of action on the viral life cycle and their ability to penetrate the blood–brain barrier [5,31]. Human microglia cells infected with the HIV_SF162_ strain (1 ng/mL) for seven days were co-exposed with 10 μM of the antiviral nucleoside reverse transcriptase inhibitor (NRTI) emtricitabine (FTC) or abacavir (ABC), the protease inhibitors lopinavir (LPV) or atazanavir (ATV), or the integrase inhibitor raltegravir (RGV), with or without siBeclin1 (4 μg). Forty-eight hours post-treatment, the viral titer was detected by HIV-1 p24 Gag protein ELISA and presented as a fold change from the control (Figure 1A). Exposure with FTC and ABC showed a 2.7- and a 3.4-fold reduction in the viral titer, respectively, while the co-administration of siBeclin1 showed minimal interactions with either antiretroviral (Figure 1A). Exposure with LPV and ATV caused a 1.3- and a 1.7-fold decrease in the viral titer, respectively, while the co-administration of siBeclin1 with LPV caused a significant decrease of 2.7-fold and the co-administration of siBeclin1 with ATV caused a significant decrease of 5.4-fold when compared to antiretroviral alone (Figure 1A). Exposure with RGV caused a 2.0-fold decrease in the viral titer, while the co-administration of siBeclin1 caused a further decrease of 2.4-fold (Figure 1A). Since antiretrovirals are commonly prescribed in combinations, we determined the response of siBeclin1 when co-administered with emtricitabine, ritonavir, and atazanavir (cART-1) or lopinavir, abacavir, and raltegravir (cART-2) in HIV-induced inflammatory cytokines and chemokines (Figure 1B). In human microglia, the exposure to cART-1 significantly decreased the secretion of IL-6, while a co-administration with siBeclin1 further diminished the IL-6 production by 1.7-fold. Similarly, cART-2 exposure significantly decreased the secretion of MCP-1 and RANTES, whereas the siBeclin1 co-administration further lessened the chemokines production by 1.8- and a 1.5-fold, respectively (Figure 1B). In HIV-infected human astrocytes, exposure to cART-1 and cART-2 significantly decreased RANTES secretions, whereas the administration of siBeclin1 further reduced RANTES release by 1.4-fold when combined with cART-1 and by 1.2-fold when combined with cART-2 (Figure 1B). This suggests that siBeclin1 interacts better with antiretrovirals that are protease inhibitors and integrase inhibitors. Overall, the data provided convincing evidence that siBeclin1 can attenuate viral-induced inflammatory responses and further reduce viral production when co-administered with antiretroviral drugs and, most importantly, with minimum cytotoxicity.

### 3.2. PEI-Man Targets Human Microglia and Human Astrocytes-Expressing Mannose Membrane Receptors and Does Not Exert Toxicity to Human Neuronal Cells

**Figure 2:**
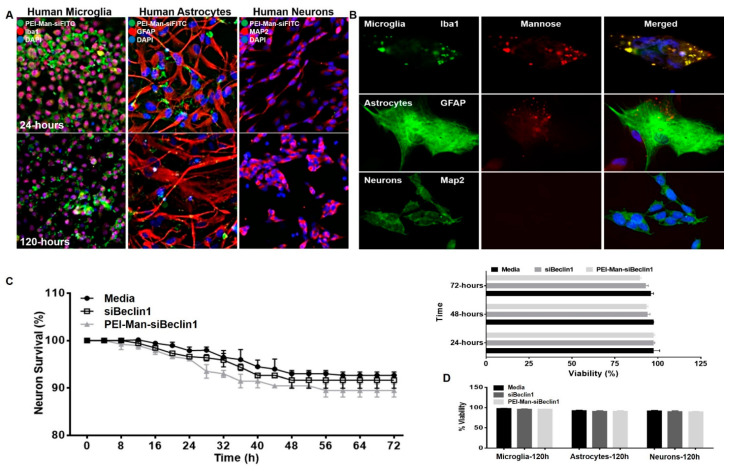
Target specificity in glial cells expressing mannose receptors. Representative immunofluorescent images of human microglia, human astrocytes, and human neurons transfected with polyethylenimine-mannose-small interfering fluorescein isothiocyanate (PEI-Man-siFITC) (green) and immunolabeled with the antibody against ionized calcium-binding adaptor molecule 1 (Iba1) (red, left panel), glial fibrillary acidic protein (GFAP) (red, middle panel), and microtubule-associated protein 2 (MAP2) (red, right panel), respectively, after 24 h (top panel) and 120 h (bottom panel) of transfection. 4′,6-Diamidino-2-phenylindole (DAPI) was used to label nuclear DNA (blue) (**A**). Representative immunofluorescent images of human microglia (green, top panel), human astrocytes (green, middle panel), and human neurons (green, bottom panel) immunolabeled with antibodies against Iba1, GFAP, and MAP2, respectively, and an antibody against the mannose receptor (red, middle panel). 4′,6-Diamidino-2-phenylindole (DAPI) was used to label nuclear DNA (blue) (**B**). Images were acquired using an inverted fluorescence microscope with a 560 Axiovision camera using 40× magnification (Zeiss). Individual neurons were assessed for survival using time-lapse imaging at the indicated time points following the indicated treatments over 72 h (**C**). Neuronal viability was confirmed using a live/dead cell fluorescence assay and manually quantified following the indicated treatments at 24, 48, and 72 h. Viability was measured by fluorescence after 120 h post-treatments (**D**). Error bars show the SEM for three independent experiments, with at least 50 cells per experiment.

Polyethyleneimines (PEIs) are positively charged polymers used to deliver siRNAs for the induction of RNA interference (RNAi) and to mediate their endosomal release [32]. Since myeloid (macrophages and microglia) and astrocytes express mannose receptors [33,34], we used a mannose-bearing PEI as a homing ligand for the specific and selective recognition of brain cells expressing the mannose receptor. Human microglia, human astrocytes, and human neuronal cell cultures were individually transfected with FITC-labeled control siRNA using a mannose (Man)-conjugated PEI reagent. After 24- and 120-h post-transfection, cells were fluorescently co-immunolabeled for the markers Iba1 for microglia (Figure 2A—left panel), GFAP for astrocytes (Figure 2A—middle panel), MAP2 for neurons (Figure 2A—right panel), and DAPI was used for the nucleus. Twenty-four hours post-treatment, green labeling was detected in microglia and astrocytes, illustrating the target specificity of PEI-Man-siFITC in glial cells but not in neurons (Figure 2A). Cells analyzed after 120 h (bottom panel) were transfected twice (at time 0 and time 48 h) and showed green fluorescence in glial cells but not in neurons (Figure 2A). The expression levels of mannose receptors were further analyzed in human microglia (Figure 2B—top panel), human astrocytes (Figure 2B—middle panel), and human neurons (Figure 2B—bottom panel) by immunofluorescence, and the representative images showed detection of the mannose receptors by microglia and astrocytes but not by neurons (Figure 2B), confirming that the binding of PEI-Man-siFITC was specific to glial cells and not to neurons. The toxicity of PEI-Man-siBeclin1 NP was analyzed in neuronal cells for up to 72 h, using a time-lapse digital methodology (Figure 2C). Tracking individual neuronal cell death using time-lapse imaging revealed no significant toxicity to neurons throughout the treatment period when compared with the media (control) and siBeclin1 alone (Figure 2C). Additionally, the cell viability of the microglia, astrocytes, and neurons were detected with fluorescence after 120-h exposure with siBeclin1 alone and PEI-Man-siBeclin1 (Figure 2D). Overall, this data provided evidence that the PEI-Man reagent can be delivered specifically to glial cells with minimal toxicity to the glial cells and neurons.

### 3.3. Biodistribution of the PEI-Man-siBeclin1 Nanoparticle in C57BL/6 Mice Brains after Intranasal Delivery

**Figure 3:**
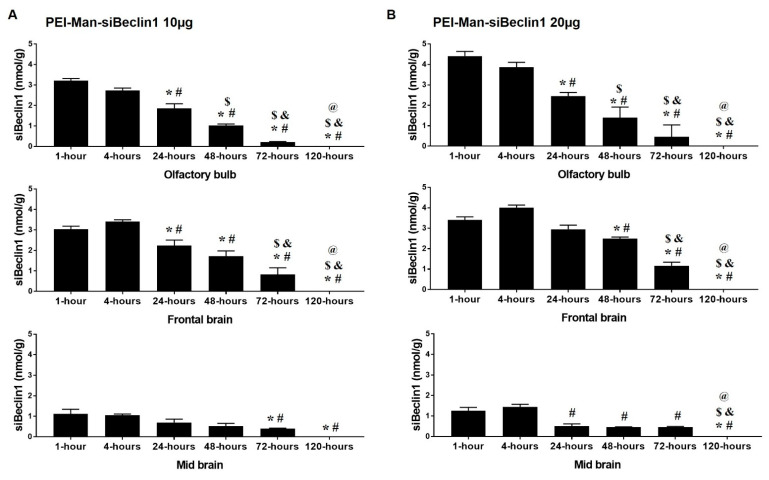
Quantitative measurements of small interfering RNA (siRNA) after intranasal delivery to C57BL/6 mice. After administration with PEI-Man-small interfering Beclin1 (siBeclin1) at 10 μg (**A**) and 20 μg (**B**), and at indicated time points, postmortem brain regions were minced, and RNA was used to measure siBeclin1 concentrations by stem-loop RT-PCR. The results are expressed in concentrations (nmol/g). Values were determined from the siBeclin1 standard curves and are presented as the mean ± the SEM of three independent experiments. (*p* < 0.05; * vs. 1 h, # vs. 4 h, $ vs. 24 h, & vs. 48 h, and @ vs. 72 h).

PEI-Man-siBeclin1 NPs and PBS (control) were delivered via the intranasal (I.N.) route to the brains of adult C57BL/6 mice. After 4, 24, 48, 72, and 120 h post-treatment, the kinetics of siBeclin1 were measured in the olfactory bulb (OB), and in different brain regions (Figure 3 and Supplementary Figure S1), by stem-loop RT-PCR. The concentrations of siBeclin1 were calculated based on a known siRNA standard curve. PEI-Man-siBeclin1 delivered at a concentration of 8 μg recovered about 2.15-nmol/g RNA at one hour and 3.20-nmol/g RNA at four hours post-delivery in the olfactory bulb and 1.15-nmol/g RNA at four hours post-delivery in the midbrain (Supplementary Figure S1). siRNA concentrations decreased at 24 h and were undetectable at 120 h post-delivery (Supplementary Figure S1). PEI-Man-siBeclin1 delivered at a concentration of 10 μg recovered about 3.05-nmol/g RNA at one hour and 3.40-nmol/g RNA at four hours post-delivery in the frontal brain (Figure 3A). At 48 h, about 1.72-nmol/g siBeclin1 was detected, and the concentration of the siRNA subsequently decreased with time. In the midbrain, the levels of siBeclin1 varied from 1.1 nmol/g at one hour to 0.40 nmol/g at 72 h post-delivery, while the detection decreased by eight-fold at 120 h post-delivery as compared with the early time points (Figure 3A). The maximal levels of siRNA detection were achieved after the PEI-Man-siBeclin1 delivery at a concentration of 20 μg NPs (Figure 3B). siRNA at concentrations of 3.42 nmol/g were detected at one hour and about 4.00 nmol/g were detected at four hours post-delivery to the frontal brain. RNA at concentrations of 1.25 nmol/g were detected at one hour and, of 1.44 nmol/g, were detected at four hours post-delivery in the midbrain (Figure 3B). In the lungs (Supplementary Figure S2), approximately 0.8 nmol/g of siBeclin1 was detected after one hour post-administration and approximately 0.18 nmol/g at four hours post-administration (Supplementary Figure S2). The binding efficiency of PEI-siBeclin1 was previously assessed by Raman spectroscopy [24], and the size, charge, and polydispersity (PDI) of the PEI-Man-siBeclin1 was determined by dynamic light scattering (DLS) using a Zeta Potential Analyzer (Malvern Instruments) (Supplementary Figure S2). The particle size and zeta potential of Pei-Man-siBeclin1 was about 22 nm and 31 mV, respectively. The PDI was less than 0.4. Since PEI does not contain a fluorochrome, the polyplex was capped with gold (Au) particles, and the morphology, shape, and structure of the PEI-Man-siBeclin1-Au NPs were analyzed by transmission electron microscopy (TEM) using a Philips 201 (Supplementary Figure S2). Both PEI-Man-Au and PEI-Man-siBeclin1-Au showed a spherical morphology with no apparent agglomeration and a particle size of about 50 nm (Supplementary Figure S2). Taken together, the data quantitatively confirms the biodistribution of siBeclin1 in the frontal and midbrain after 1, 4, 24, 48 h of intranasal delivery, with a significant decrease in RNA detection at 72 and 120 h post-delivery.

### 3.4. PEI-Man-siBeclin1 Nanoparticle Delivered to the Brain Causes Minimal Toxicity and Reduces the Secretion of Inflammatory Molecules

**Figure 4:**
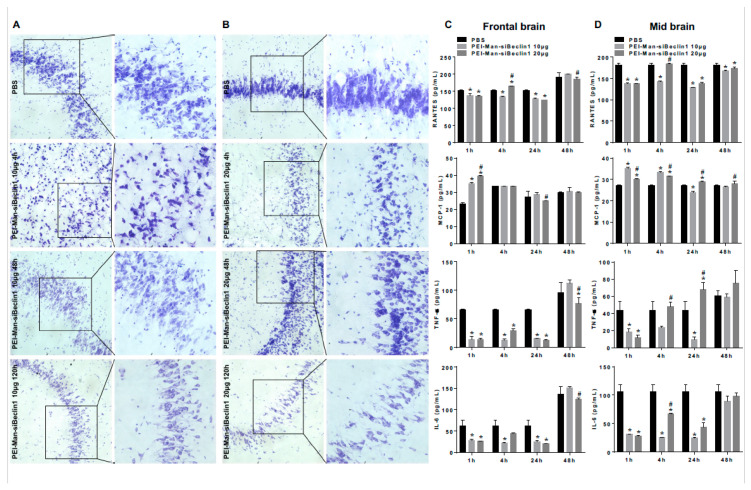
Histological and biochemical analyses of postmortem brain regions after the intranasal delivery of PEI-Man-siBeclin1 in C57BL/6 mice. Representative images of the Nissl staining of adult mice brains removed postmortem after treatment with phosphate-buffered saline (PBS) (**A**,**B**, top panel) or PEI-Man-siBeclin1 at 10 μg (**A**) and 20 μg (**B**) at 4, 48, and 120 h post-treatment. Corresponding tissues at 1, 4, 24, and 48 h were minced and used to detect RANTES, MCP-1, tumor necrosis factor alpha (TNF-α), and IL-6 by ELISA (**C**,**D**). (**A**,**B**) Images were acquired using an inverted fluorescence microscope with a 560 Axiovision camera using 20× and 40× magnification (Zeiss). (**C**,**D**) Values were determined from the standard curves and are presented as the mean ± the SEM of three independent experiments (*p* < 0.05; * vs. PBS and # vs. 10 µg).

Brains recovered at necropsy were stained by the Nissl method to identify the neuronal morphology and approximate any changes in the neuronal number within the brain area of interest (prefrontal cortex) (Figure 4A,B and Supplementary Figure S3). After 30 min and 48 h, no noticeable changes in the neuronal morphology and tissue integrity were detected in postmortem brains exposed to PEI-Man-siBeclin1 at 8 μg, when compared with PBS-administrated mice (Supplementary Figure S3). Homogenized brain tissues recovered at 48 h post-treatment were used to measure the proinflammatory molecules and showed no differences in MCP-1, RANTES, IL-6, and TNF-α secretion when compared to the PBS-administrated mouse cohort (Supplementary Figure S3). A lack in the secretion of inflammatory molecules, along with the absence in morphological changes, correlated with a lack of adverse behavioral effects in the motor (Supplementary Figure S4). Likewise, the prefrontal cortex from postmortem brains exposed to PEI-Man-siBeclin1 administered at 10 μg (Figure 4A) and 20 μg (Figure 4B) concentrations showed a well-maintained neuronal morphology with no evidence of neuron swelling or vacuolation at 4, 48, and 120 h post-intranasal delivery. Furthermore, there were no apparent signs of gliosis or neuronal loss in the tissues exposed to PEI-Man-siBeclin1 at 10 μg and 20 μg concentrations at 4, 48, and 120 h post-delivery when compared to the tissues exposed to PBS (Figure 4A,B). We also detected for gliosis by immunofluorescence staining using postmortem brain tissues at 24-h after intranasal delivery of PBS and PEI-Man-siBeclin1 at 20 μg. No visible difference in fluorescence intensity was detected in tissues treated with PEI-Man-siBeclin1 versus PBS (Supplementary Figure S5). The other half of the brain recovered at necropsy was homogenized after 1, 4, 24, and 48 h post-treatment and showed no significant changes in the secretion of the chemokines RANTES and MCP-1 in the frontal cortex and in the midbrain after extended time points (Figure 4C,D). However, a significant decrease in both IL-6 and TNF-α were detected in homogenates extracted from the frontal cortex and the midbrain when compared to PBS-treated brains (Figure 4E,F). This finding is of great interest, as these cytokines are heavily associated with the inflammatory effects of HIV—in fact, elevated levels of IL-6 and TNF-α—and are often detected in HIV-encephalitic brains [35]. The cytokine and chemokine levels were also analyzed at later time points (data not shown); however, only time points that showed high detections of siRNA after intranasal brain delivery were considered. The overall findings revealed no noticeable differences between the neuronal structure tissue integrity, with significant changes in the secretion of proinflammatory molecules in PEI-Man-siBeclin1 NPs administered intranasally when compared to PBS-treated brains.

### 3.5. Biodistribution of DIR-Liposome-Nanoparticles in C57BL/6 Mice after Intranasal Delivery

**Figure 5:**
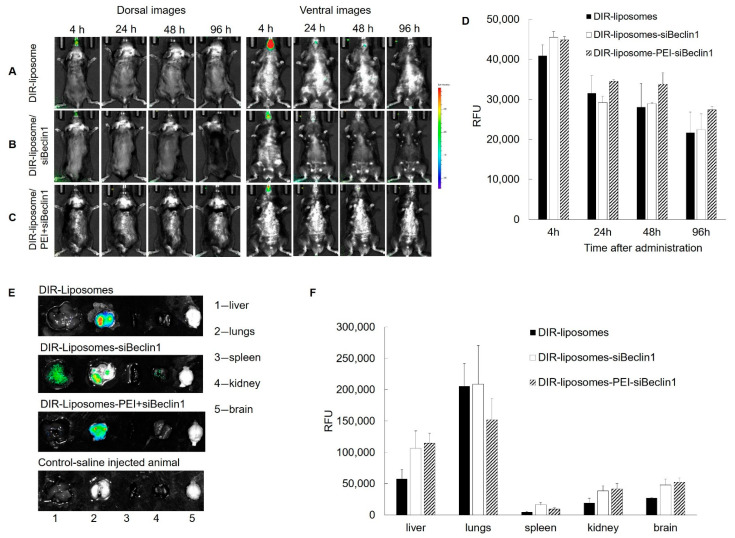
Live imaging analysis of DIR-labeled nanoparticles after intranasal administration in C57BL/6 mice. Liposomes labeled with a fluorescent hydrophobic dye, DIR, were injected into C57BL/6 mice intranasally alone (**A**), or loaded with siRNA (**B**), or loaded with the PEI-siRNA complex (**C**) (3 × 10^12^ particles/mL), and the brain accumulation of liposome-siRNA formulations was imaged by the In Vivo Imaging System (IVIS) up to 96 h (**D**). Fluorescent accumulation was quantified in different organs recovered at necropsy (**E**,**F**).

The next study combined the properties of PEI-Man and lipid systems for siRNA delivery. Liposomes act as transfection reagents, with some lipids showing increased endocytosis and influencing endosomal escape [32]. Another advantage of the lipidation of polyplexes is that it generally decreases their toxicity [36]. To further confirm the biodistribution of siBeclin1, we used a fluorophore-labeled anionic liposome (LP) encapsulated with PEI-Man-siBeclin1 NPs (LP-NPs) and detection by the In vivo Imaging System (IVIS) (Figure 5). DIR-liposomes were injected into C57BL/6 mice intranasally alone (Figure 5A), loaded with siRNA (Figure 5B), or loaded with a PEI-siRNA complex (Figure 5C) and imaged by IVIS up to 96 h. High levels of DIR-LP-NPs were recorded throughout the duration of the experiment (up to 96 h), while the highest fluorescent detection was observed at four hours in mouse brains after intranasal administration (Figure 5A–D). Mice were sacrificed at the endpoint (96 h), perfused to eliminate the blood, and the main organs were imaged by IVIS (Figure 5E,F). The quantification of the fluorescence levels at necropsy suggested that the fluorescence signals of the DIR-LP-NPs decreased in a row: lungs > liver > kidney ≅ brain > spleen (Figure 5E). For each group of NPs, the greatest signal was detected in the lungs, although a considerable signal was also seen in the brain (Figure 5E). This data confirms the biodistribution of the siBeclin1 NPs in mice after intranasal administration.

## 4. Discussion

This present study explored the potential use of siBeclin1 as an autophagy-based adjunctive therapy for HIV in myeloid and astrocytic cells and confirmed the delivery of siBeclin1 NPs in the brain via the intranasal route. To this day, great efforts are still directed in identifying a potential complementary therapy for the HIV disease and HIV-mediated neurocognitive disorders. Despite the success of cART, several limitations remain to be addressed, including poor BBB penetrance, drug toxicity, and the inability to eliminate long-term chronic inflammation [5,37,38]. Autophagy is a catabolic pathway that engulfs and sequesters cytoplasmic proteins in double-membrane-bound autophagosomes and are delivered to the lysosome for degradation [39]. Beclin1, in a complex with the class III phosphatidylinositol 3-kinase Vps34, is essential for the formation of the isolation membrane of the autophagosome [40]. Modulation of the autophagy pathway has been considered as a therapeutic target for several diseases, including Alzheimer’s disease, cardiac diseases, and cancer [41,42,43,44]. However, the relation between autophagy and pathogens is extremely complex, and their interactions differ between cell types. In terms of HIV, modulating autophagy has been proposed as a potential alternative approach to reduced HIV infection [45,46,47,48,49]. A study reported that several autophagy inducers extended the retention and sustained release of long-acting antiretroviral nanoformulations in HIV-infected human monocyte-derived macrophages [50]. The authors suggested that the sustained release is due to the formation of autophagosomes that enable the intracellular retention of antiretroviral nanoformulations. On the other hand, we have shown that reduced levels of Beclin1 in glial cells (both microglia and astrocytes) lessen HIV infection and inflammatory molecules in vitro [51,52,53]. Thus, we propose the use of siBeclin1 as an adjunctive therapy, in combination with cART, to reduce the HIV titer and to reduce the secretion of HIV-induced inflammatory molecules. Here, we demonstrated that siBeclin1 can be used to increase the efficacy of antiretrovirals in decreasing viral infections and that siBeclin1 can also be used to reduce the secretion of inflammatory cytokines such as, MCP-1, IL-6, and RANTES in glial cells (Figure 1). Overall, the antiviral response of LPV, ATV, and RGV was significantly enhanced when Beclin1 was inhibited, indicating that they are Beclin1-dependent. Furthermore, ATV and RGV are protease inhibitors that inhibit proteolytic cleavage by enzymes released by HIV, suggesting that Beclin1 might interact with the protease molecule or the substrate-binding domain on the protease. The interaction between Beclin1, the protease, and the integrase inhibitors was further illustrated in the anti-inflammatory responses in combination with antiretrovirals. In general, silencing Beclin1 had a greater response in reducing MCP-1 in human microglia when compared to human astrocytes. While we do not know the exact reason, it could be related to the higher concentrations of MCP-1 secreted in astrocytes, and, thus, higher concentrations of siRNA might be needed. Secondly, cART-1 was very effective in reducing MCP-1 and RANTES in HIV-infected human microglia, and further reduction with siBeclin1 was not detected. On the other hand, cART-2 had a milder effect and did show further reduction with siBeclin1. Cytokine production could reflect the amount of viral titer in the infected cell. In fact, the protease inhibitor LPV showed a mild efficacy on the inhibition of viral replication (Figure 1A), and cART-2 included LPV. The transcription factor NF-kB regulates the genes for cytokines, and while we did not measure the activation of NF-kB in this study, we have done so previously and showed a significant downregulation of NF-kB with siBeclin1 [53]. 

The introduction of RNAi-based therapeutics faced several difficulties, such as low stability, toxicity, and off-target effects [54,55,56]. However, improvements in design and delivery approaches have advanced several RNAi-based therapeutics to clinical trials [57,58]. One of the main challenges in the development of RNAi-based therapeutics is to maintain RNA molecule stability during delivery in vivo. In this study, in order to protect siRNA from degradation, the nucleotide was encapsulated with a mannosylated polyethyleneimine polymer. We have previously shown a significant reduction in the expression levels of the protein Beclin1 with minimum toxicity in murine brains administered with siBeclin1 [24]. Here, we showed the specificity of the PEI-Man-siBeclin1 NPs to target microglia and astrocytes without targeting neurons. This is very important, as HIV infects the glia and are the main cell type secreting inflammatory molecules that can lead to neuronal injury and cell death. Furthermore, neurons are postmitotic cells and heavily dependent on the autophagy pathway for the clearance of protein aggregates, misfolded proteins, and dysfunctional organelles [59]. Thus, prolonged compromised autophagy in neuronal cells can lead to neurodegenerative diseases [60,61,62]. Since autophagy is an essential pathway for neuronal homeostasis and survival, we have modified our previously reported nanoformulation, which consisted of PEI-siBeclin1, with the addition of mannose molecules to specifically target mannose receptor-expressing glial cells. The mannose receptor is a transmembrane glycoprotein from the C-type lectin family that can be used as a potential surface target for drug delivery using nanocarriers [63,64,65]. This receptor is expressed in a wide range of cells, including macrophages, dendritic cells, and, as mentioned earlier, glial cells [66,67]. Interestingly, a study showed the direct involvement of the mannose receptor in an HIV infection of astrocytes [68]. Here, following the transfection of PEI-Man-FITC, we were able to detect the colocalization of FITC with the fluorescently immunolabeled cellular markers GFAP in astrocytes and with Iba1 in the microglia but not with MAP2 in the neurons (Figure 2). This suggests the specificity of our NP for mannose-expressing glial cells among brain cells. Accordingly, we confirmed the expression of the mannose receptor in the astrocytes and microglia, while showing that the neurons lacked the receptor (Figure 2). In addition, we showed that the presence of PEI-Man-siBeclin1 did not elicit neuronal cell death in vitro (Figure 2). The ability to selectively target the microglia and astrocytes is extremely useful for treating chronic inflammatory diseases. Reports have suggested targeting astrocytes to improve the functional outcome following a stroke [69] and the responses after a brain insult or injury [70,71]. Additionally, a study showed that microglia were selectively labeled using quantum dots in primary cortical cultures and the brain due to the mannose receptors present on the surface of the microglia [72]. However, there is no sufficient evidence of using the approach of targeting glial cells to diminish HIV in the brain. The characterization of PEI-Man-encapsulated siBeclin1 NPs showed a spherical morphology of the NPs, with an average size of 50 nm and a positive zeta potential (Supplementary Figure S2). After intranasal delivery, the highest concentrations of siBeclin1 were detected at 1, 4, 24, and 48 h in frontal brain tissues, while concentrations of the siRNA were drastically reduced at 72 and 120 h (Figure 3). Interestingly, concentrations of siBeclin1 were also detected in the midbrain sections at one and four hours post-delivery. The live imaging analysis further confirmed the brain accumulation of NPs after 4, 24, 48, and 96 h post-administration (Figure 5). This time-frame coincided with the observed downregulation of Beclin1 protein expression levels in the postmortem brain tissues of mice intranasally administered with PEI-siBeclin1 previously reported by us [24]. Other reports using the intranasal administration of siRNA as a treatment strategy to acutely suppress the serotonin 5-HT1A autoreceptor to evoke marked antidepressant-like effects in mice detected siRNA in the brain 48 h post-intranasal delivery [73]. Moreover, Renner et al. also reported the distribution of fluorescently labeled siRNA to the olfactory bulbs of mice via the olfactory nerve pathway as early as 30 min following intranasal administration [74]. Similarly, a study using a rat model of focal cerebral ischemia showed that intranasally administered siRNA targeting high mobility group box 1 (HMGB1) can be delivered within an hour and distributed widely in the brain. Gene knockdown persisted for 24 h, alleviating the neurological and behavioral deficits in the postischemic rat brain [75]. Coinciding with these studies, here, we reported that PEI-Man-siBeclin1 NPs can be delivered to the mouse brain via the intranasal route without noticeable evidence of the tissue toxicity and no significant secretion of the inflammatory molecules (Figure 4). Furthermore, while assessing the loss in motor function as an indicator of neuronal dysfunction, mice that were intranasally administrated with PEI-Man-siBeclin1 showed no significant impairment in motor coordination when compared to PBS-treated mice (Supplementary Figure S4), further confirming the noninvasive and nontoxic properties of PEI-Man-siBeclin1 NPs.

In conclusion, we reported the potential use of targeting the autophagy protein Beclin1 as an effective adjunctive therapy in combination with antiretrovirals for the attenuation of HIV infection and HIV-induced inflammatory molecules in HIV-infected glial cells (in vitro). We also provided both qualitative and quantitative evidence of brain delivery, along with myeloid target specificity, via a noninvasive intranasal delivery of PEI-Man-siBeclin1 NPs. This potential therapeutic approach could selectively decrease autophagy levels in microglia and astrocytes, with minimum alterations to neuronal autophagy in the brain. Current studies are examining the efficacy of the NPs using an HIV-infected mouse model.

## Data Availability

Data is contained within the article or supplementary material.

## Supplementary Materials

The following are available online at https://www.mdpi.com/1999-4923/13/2/223/s1: Figure S1: Quantitative measurements of siRNA after intranasal delivery to C57BL/6 mice. Figure S2: Characterization of PEI-siBeclin1. Figure S3: Histological and biochemical analyses of postmortem brain regions after the intranasal delivery of PEI-Man-siBeclin1 8 μg in C57BL/6 mice. Figure S4: Behavioral assessment after PEI-Man-siBeclin1 8 μg intranasal delivery in mice. Figure S5: Gliosis evaluation after intranasal administration in C57BL/6 mice.
