# Supplementary material for: Targeting Beclin1 as an Adjunctive Therapy against HIV Using Mannosylated Polyethylenimine Nanoparticles
Source: Pharmaceutics. 2021 Feb 6;13(2):223. doi: 10.3390/pharmaceutics13020223 (PMC7915950; doi:10.3390/pharmaceutics13020223)
Supplement: Supplementary file 1 [file pharmaceutics-13-00223-s001.pdf]

pharmaceutics

Supplementary Materials: Targeting Beclin1 as an Adjunctive Therapy against HIV Using Mannosylated Polyethylenimine Nanoparticles

Myosotys Rodriguez, Yemmy Soler, Mohan Kumar Muthu Karuppan, Yuling Zhao, Elena V. Batrakova and Nazira El-Hage

Citation: Rodriguez, M.; Soler, Y.; Muthu Karuppan, M.K.; Zhao, Y.; Batrakova, E.V. El-Hage, N. Targeting Beclin1 as an Adjunctive Therapy against HIV Using Mannosylated Polyethylenimine Nanoparticles. *Pharmaceutics* **2021**, *13*, x. https://doi.org/10.3390/xxxxx

Academic Editor: Thierry Vandamme
Received: 29 December 2020
Accepted: 31 January 2021
Published: date

Publisher's Note: MDPI stays neutral with regard to jurisdictional claims in published maps and institutional affiliations.

Copyright: © 2021 by the authors. Submitted for possible open access publication under the terms and conditions of the Creative Commons Attribution (CC BY) license (http://creativecommons.org/licenses/by/4.0/).

Supplementary material S1

Materials And Methods

Characterization of PEI-siBeclin1

Electrical potential, average uniformity, and average size of the NPs were determined by Zeta potential and Polydispersity index (PDI). Dynamic light scattering was measured using a Nano ZS90 Zetasizer (Malvern Instruments) as previously performed [1]. For Transmission electron microscopy (TEM) imaging purposes only, PEI-Man-polyplex with and without encapsulated siBeclin1 were capped with gold nanoparticles (AuNPs). Images were acquired using a Phillips CM-200 200 kV transmission electron microscope operated at 80 kV.

Behavior assessment

After intranasal administration, behavioral tests assessing motor skills were performed. On the day of behavior testing, mice were habituated in the testing room for one hour prior to assessment. Mice were allowed 3 attempts for each behavior test with a 5-minute time interval between trials and the best score recorded. The rotor rod is an elevated rotating rod which evaluates coordination and endurance. Mice were placed on the rod without prior training and the rod accelerated from 1 to 40 rpm in 1.0 rpm stages per 15 seconds. Latency to fall to was recorded in seconds. The grip strength test assesses muscle weakness based on the instinctual tendency of mice to grasp objects with its forelimbs. Mice were placed on a computerized grid connected to a force gauge meter and once grasping the grid, steadily pulled on by the tail to measure force. Test were repeated 5 times in 30 second intervals with the highest tension value being used as measurement of strength. Peak grip was normalized to body weight for each animal.. Horizontal bars are an additional measure of forelimb strength and coordination using elevated 2- and 4-mm diameter bars. Mice were tested on the ability to hold on to each bar and/or traverse the bar to the end poles. Duration of stay before falling was scored from 1–5, with 5 being the highest score for staying at least 30 seconds or reaching the end pole. Mice were tested on the 2 mm bar followed by the 4 mm bar and the two scores added.

Immunohistochemistry

Brain tissue sections were fixed in 4% paraformaldehyde, permeabilized with 0.1% Triton X-100, and blocked in 10% milk/0.1% goat serum. Sections were immunolabeled with the Glial fibrillary acidic protein (GFAP) antibody (Cat#: ab5804, Millipore, Bedford, MA, USA) at a 1:1000 dilution, and the Ionized calcium binding adaptor molecule 1 (Iba1) antibody (Cat#: sc32725, Santa Cruz Biotechnology) at a 1:100 dilution. Immunoreactivity was visualized with secondary antibodies from Molecular Probes (Carlsbad, CA, USA). 4′,6-diamidino-2-phenylindole (DAPI) was used to label cell nuclei. Images were analyzed using an inverted fluorescence microscope with a 560 Axiovision camera (Zeiss).

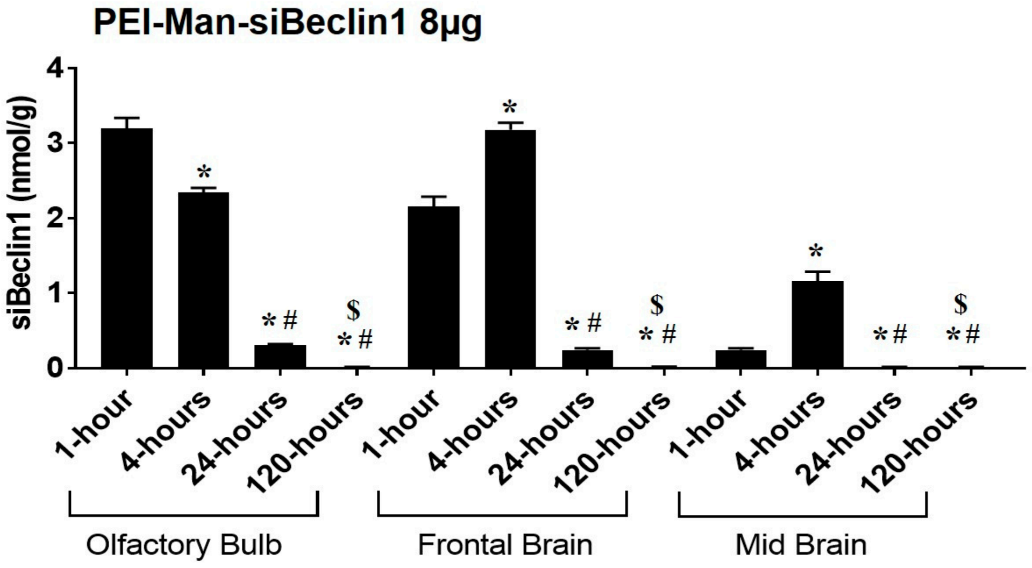

Figure S1. Quantitative measurements of siRNA after intranasal delivery to C57BL/6 mice. After administration with PEI-Man-siBeclin1 at 8 µg at indicated timepoints, postmortem brain regions were minced and used to measure siBeclin1 concentrations by Stem-loop RT-PCR. The results are expressed in concentration (nmol/g). Values were determined from siBeclin1 standard curves and are presented as the mean ± the SEM of three independent experiments. ($p < 0.05$ * vs. 1 h, # vs. 4 h, $ vs. 24 h, & vs. 48 h, @ vs. 72 h).

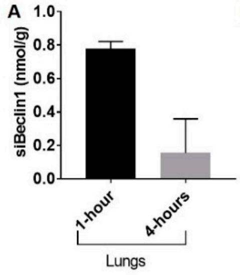

B

Sample	Size	PDI	Zeta potential (mV)
PEI-Man	-	0.442±0.10	23.6±2.41
siBeclin1	-	0.38±0.28	-17.2mV±0.58
PEI-Man-siBeclin1	21.56±8.12nm	0.337±0.41	31.1±1.63

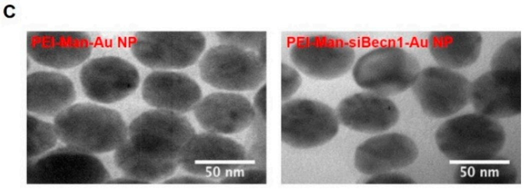

Figure S2. Characterization of PEI-siBeclin1. After administration with PEI-Man-siBeclin1 at 8 µg at 1 and 4 h, postmortem lung tissues were minced and used to measure siBeclin1 concentrations by Stem-loop RT-PCR (**A**). The results are expressed in concentration (nmol/g)**.** Values were determined from siBeclin1 standard curves and are presented as the mean ± the SEM of three independent experiments. The Zeta potential (expressed in mV), and PDI of PEI-siBeclin1 NPs were analyzed after synthesis (**B**). Synthesized NPs were analyzed by transmission electron microscopy (TEM) and showed spherical morphology with no apparent agglomeration and a particle size of about 50 nm (**C**).

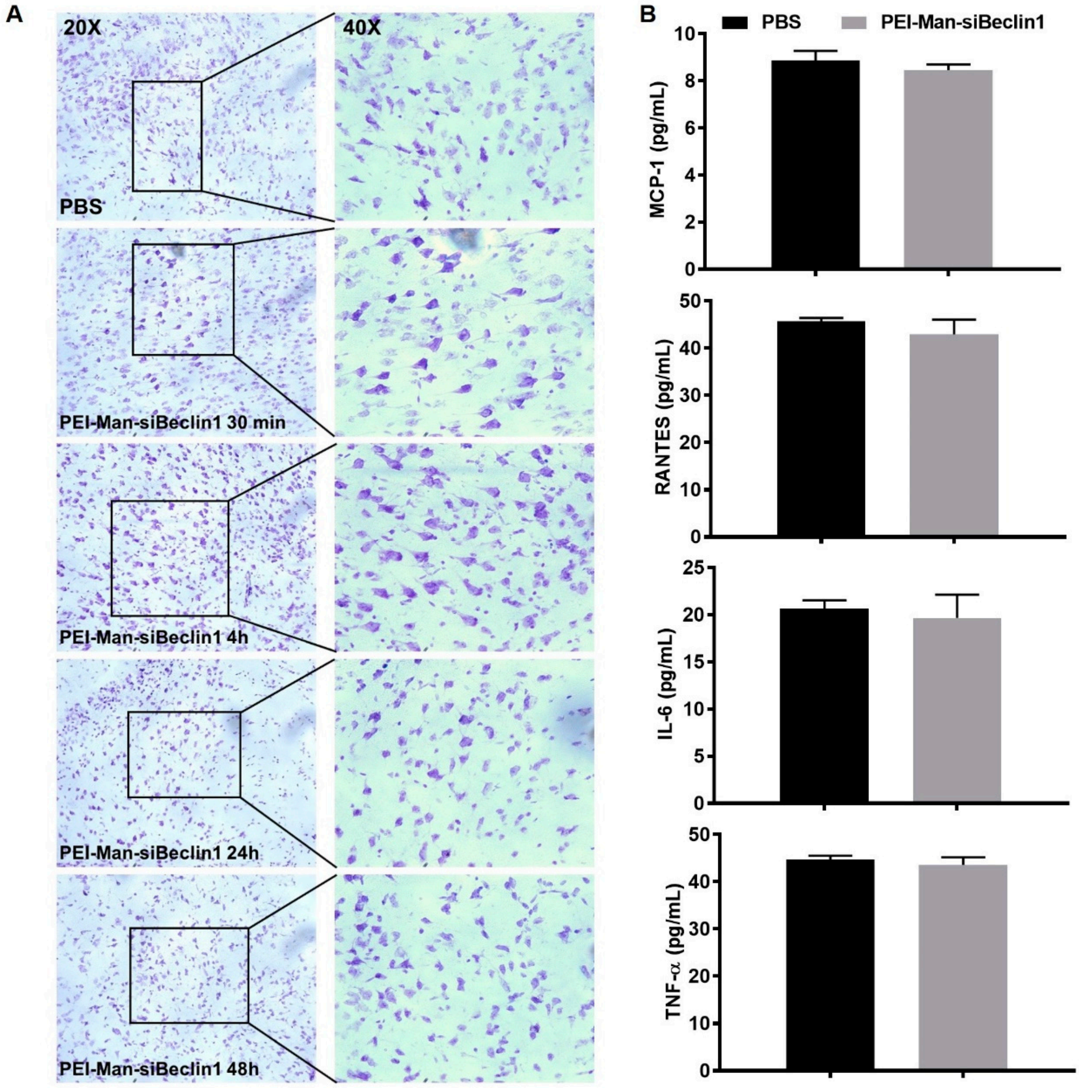

Figure S3. Histological and biochemical analyses of postmortem brain regions after the intranasal delivery of PEI-Man-siBeclin1 8 μg in C57BL/6 mice. Representative images of Nissl staining of adult C57BL/6 mice brains removed postmortem administrated with PBS, and PEI-Man-siBeclin1 8 μg at 4, 48, and 120 h (**A**). Corresponding tissues at 30 min, 4, 24 and 48 h were minced and used to detect the levels of MCP-1, RANTES, IL-6 and, TNF-α by ELISA (**B**). (**A**) Images were acquired using an inverted fluorescence microscope with a 560 Axiovision camera using 20× and 40× magnification (Zeiss). (**B**) Values were determined from standard curves and are presented as the mean ± the SEM of three independent experiments.

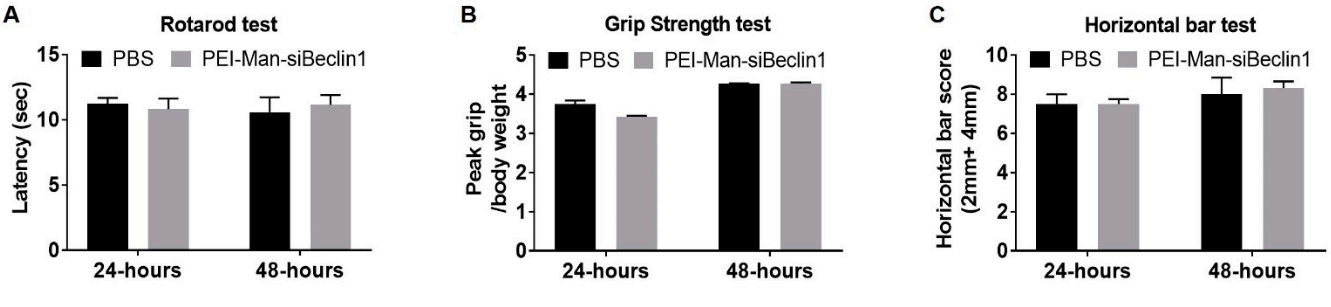

Figure S4. Behavioral assessment after PEI-Man-siBeclin1 8 μg intranasal delivery in mice. Motor skills were examined in adult C57BL/6 mice by accelerating rotarod (**A**), forelimb grip strength (**B**), and horizontal bars assessment of motor coordination (**C**) at 24 and 48 h after intranasal delivery of PBS and PEI-Man-siBeclin1 8 μg.

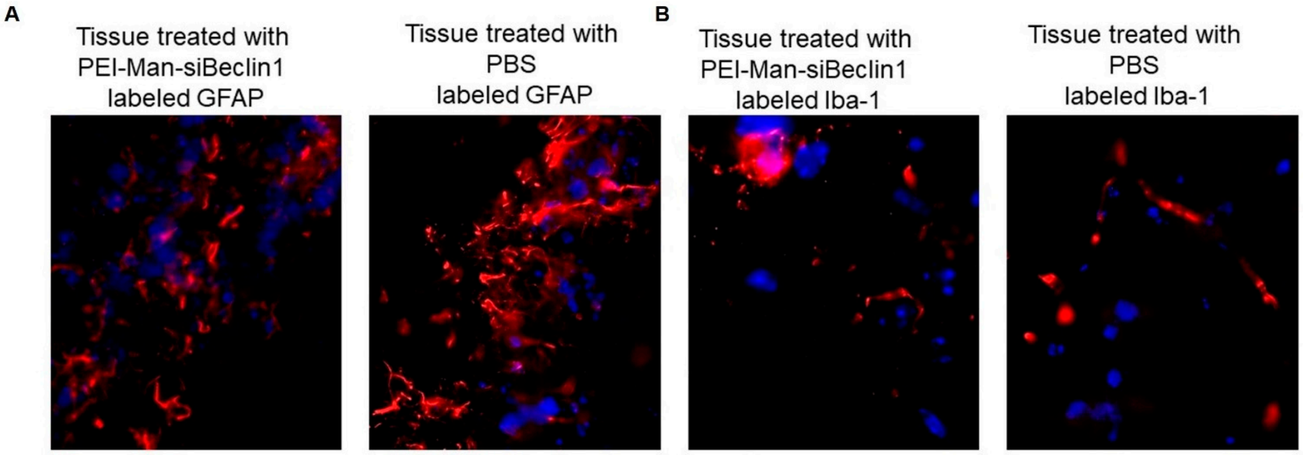

Figure S5. Gliosis evaluation after intranasal administration in C57BL/6 mice. Representative immunofluorescent images of postmortem brain tissues at 24 h after intranasal delivery of PBS and PEI-Man-siBeclin1 20 μg, immunolabeled with GFAP antibody (red) (**A**) and Iba-1 antibody (red) (**B**). Images were acquired using an inverted fluorescence microscope with a 560 Axiovision camera and a 40X magnification (Zeiss).

Reference

1. Kaushik, A.; Jayant, R.D.; Nikkhah-Moshaie, R.; Bhardwaj, V.; Roy, U.; Huang, Z.; Ruiz, A.; Yndart, A.; Atluri, V.; El-Hage, N.; et al. Magnetically guided central nervous system delivery and toxicity evaluation of magneto-electric nanocarriers. *Sci. Rep.* **2016**, *6*, 25309, doi:10.1038/srep25309.
